# Advances in metabolic modeling of oleaginous microalgae

**DOI:** 10.1186/s13068-018-1244-3

**Published:** 2018-09-05

**Authors:** Juan D. Tibocha-Bonilla, Cristal Zuñiga, Rubén D. Godoy-Silva, Karsten Zengler

**Affiliations:** 1 Grupo de Investigación en Procesos Químicos y Bioquímicos, Departamento de Ingeniería Química y Ambiental, Universidad Nacional de Colombia, Av. Carrera 30 No. 45-03, Bogotá, D.C. Colombia; 2 Department of Pediatrics, University of California, San Diego, 9500 Gilman Drive, La Jolla, CA 92093-0760 USA; 3 Department of Bioengineering, University of California, San Diego, 9500 Gilman Drive, La Jolla, CA 92093-0412 USA; 4 Center for Microbiome Innovation, University of California, San Diego, 9500 Gilman Drive, La Jolla, CA 92093-0436 USA

**Keywords:** Oleaginous phototrophs, Lipid production, Constraint-based metabolic modeling, Central carbon metabolism

## Abstract

Production of biofuels and bioenergy precursors by phototrophic microorganisms, such as microalgae and cyanobacteria, is a promising alternative to conventional fuels obtained from non-renewable resources. Several species of microalgae have been investigated as potential candidates for the production of biofuels, for the most part due to their exceptional metabolic capability to accumulate large quantities of lipids. Constraint-based modeling, a systems biology approach that accurately predicts the metabolic phenotype of phototrophs, has been deployed to identify suitable culture conditions as well as to explore genetic enhancement strategies for bioproduction. Core metabolic models were employed to gain insight into the central carbon metabolism in photosynthetic microorganisms. More recently, comprehensive genome-scale models, including organelle-specific information at high resolution, have been developed to gain new insight into the metabolism of phototrophic cell factories. Here, we review the current state of the art of constraint-based modeling and computational method development and discuss how advanced models led to increased prediction accuracy and thus improved lipid production in microalgae.

## Background

Photosynthetic microorganisms have been recognized as one of the oldest life forms on Earth [1]. These organisms, including microalgae such as *Chlamydomonas* sp., *Synechocystis* sp., and *Chlorella* sp., have attracted significant attention from the biotechnology industry because of their ability to efficiently transform renewable resources (CO_2_, light, and water) into biomass and fuel precursors [2]. The photosynthetically produced biomass along with accumulated and secreted metabolites can be employed for the downstream synthesis of fuels (e.g., ethanol, biodiesel, and biocrude) and fine chemicals (e.g., pigments and organic acids) [3].

The world’s ever-expanding requirement for cheap energy and fuel requires constant improvement of production platforms to meet the demand. The increased fuel consumption has led to an increase in global greenhouse gas emissions [4], exemplified by a sharp increase in CO_2_ levels from 280 ppm before the industrial revolution to today’s 407 ppm [5, 6]. Over 75% of these CO_2_ emissions have been attributed to the burning of fossil fuels [7, 8], rendering the reduction of humanity’s carbon footprint a major global technological challenge. One alternative to address this challenge is increased utilization of biofuels from renewable resources and thus significant efforts have been underway to improve the efficiency of production of various biofuels [9].

Biofuels are categorized into first-, second-, and third-generation biofuels depending on the type of raw material that is used for their production [10]. First-generation biofuels are produced from agricultural crops; one example being bioethanol production from sugarcane. These biofuels have been widely criticized as they pose extra demands on food production, which consequently raises food prices. Additionally, intensive agricultural processes to satisfy cost-effective production of crops for biofuels can lead to eutrophication and contamination of environmental resources [8, 11, 12]. As an alternative second-generation biofuels generated from woody waste and inedible food parts, such as biofuels from lignocellulosic biomass, have been proposed as a substitute for first-generation biofuels generated from food sources [10]. Secondary biofuels still require fertile land and often substantial amount of water for irrigation, limiting their areas of production. Third-generation biofuels, such as biosustainable production by microalgae, have thus been investigated to complement first- and second-generation biofuels. Third-generation biofuels also face several drawbacks which need to be overcome before turning into an economically viable alternative [13]. One of the largest challenges for third-generation biofuels from photosynthetic microorganisms lies in the harvesting process and downstream refinement of compounds of interest. For example, the costly recovery process of lipids from microalgal biomass, which in the case of biodiesel can account for up to 50% of the final cost [14], often prevents algae biofuel operations to be economically viable [14]. Higher lipid content would offset these staggering costs and would widely benefit the profitability and applicability of a third-generation biofuel technology. An early study by the US Department of Energy from 1978 reported that a lipid content of 60% would be necessary for third-generation biofuels to become economically feasible [15]. This number is now being revised to 20–40%, depending on strain and cultivation conditions [16]. Increasing the lipid content of phototrophs has thus been a major focus for the biofuel industry. Major efforts to improve lipid content have been focused on optimizing culture conditions and on advanced strain engineering designs, both strategies of which greatly benefit from the use of metabolic modeling. In this review we compare various computational methods used for the rational design of strains and culture media, including flux balance analysis (FBA), dynamic flux balance analysis (dFBA), ^13^C metabolic flux analysis (^13^C MFA), and elementary modes (EM) analysis. We focus in particular on the latest insights into central carbon metabolism (tricarboxylic acid cycle, the Calvin cycle, the glyoxylate shunt, glycolysis/gluconeogenesis, and the pentose-phosphate pathway) of oleaginous microalgae obtained by computational modeling as it is most relevant for production of biofuels and fuel precursors. Furthermore, we discuss the impact of time course modeling as well as the importance of incorporating compartmentalization into genome-scale models for microalgae and highlight the complexity of modeling lipid metabolism to increase biofuel productivity.

### Oleaginous photosynthetic microorganisms

Microalgae have historically been classified into two classes: bacterial microalgae (C*yanophyta*) and eukaryotic microalgae, the latter including green algae (*Chlorophyta*), red algae (*Rhodophyta*), and diatoms (*Bacillariophyta*). Characteristic for all microalgae is their ability to grow photoautotrophically with CO_2_ and light as only carbon and energy sources. Several microalgae are also able to grow heterotrophically in the absence of light using various organic substrates, or grow mixotrophically, which refers to the uptake of organic carbon, e.g., glucose, sucrose, or acetate during growth in the light [17]. Oleaginous microalgae are attractive cell factories for the production of third-generation biofuels due to their ability to achieve an outstanding accumulation of lipids, in some cases surpassing 20% of total biomass in dry weight [13] and reaching economic feasibility [16]. Some studies have reported microalgae lipid productivities around 136,900 L ha^−1^ year^−1^ [12], which are several times higher than those achieved by oil palm plantations (22,780 L ha^−1^ year^−1^) [12, 18]. Microalgae have also been explored for the production of non-lipid-based biofuels [12]. Several genera of microalgae have been used for biofuel production, and metabolic models now exist for organisms such as *Chlamydomonas* [19–30], *Chlorella* [31–35], *Nannochloropsis* [36–38], *Synechocystis* [39–46], *Tetraselmis* [47], *Monoraphidium* [48], *Ostreococcus* [49], *Tisochrysis* [50], and *Phaeodactylum* [51–54]. The genetic tractability of several microalgae (*Chlamydomonas*, *Synechocystis*, *Phaeodactylum*) [55] also renders them interesting for gene-knockout studies using metabolic modeling tools. Metabolic models have enabled retrieving key information about central carbon metabolism, nutrient dependence, and distribution of reactions throughout different compartments in these organisms. Furthermore, dependence of carbon allocation on nutrient availability and the differential role of the main carbon pathways under several growth conditions have been revealed using these models. Examples for these findings will be discussed in detail below.

### Metabolic modeling

Various modeling approaches have been deployed to improve the applicability of microorganisms for industrial applications. Modeling efforts can be categorized into isotope labeling-based, kinetic-based, and constraint-based approaches [56]. Isotope labeling studies and kinetic-based approaches are restricted to core metabolic networks or whole-cell analyses, although none of those methods is yet available on a genome scale and neither of these approaches considers organelle-specific compartmentalization. Constraint-based modeling approaches are currently the most widely used methods in metabolic modeling of oleaginous microalgae. These models enable in-depth understanding of microorganisms and their metabolism by simulating intracellular fluxes throughout a metabolic network, often at genome scale [57].

**Fig. 1:**
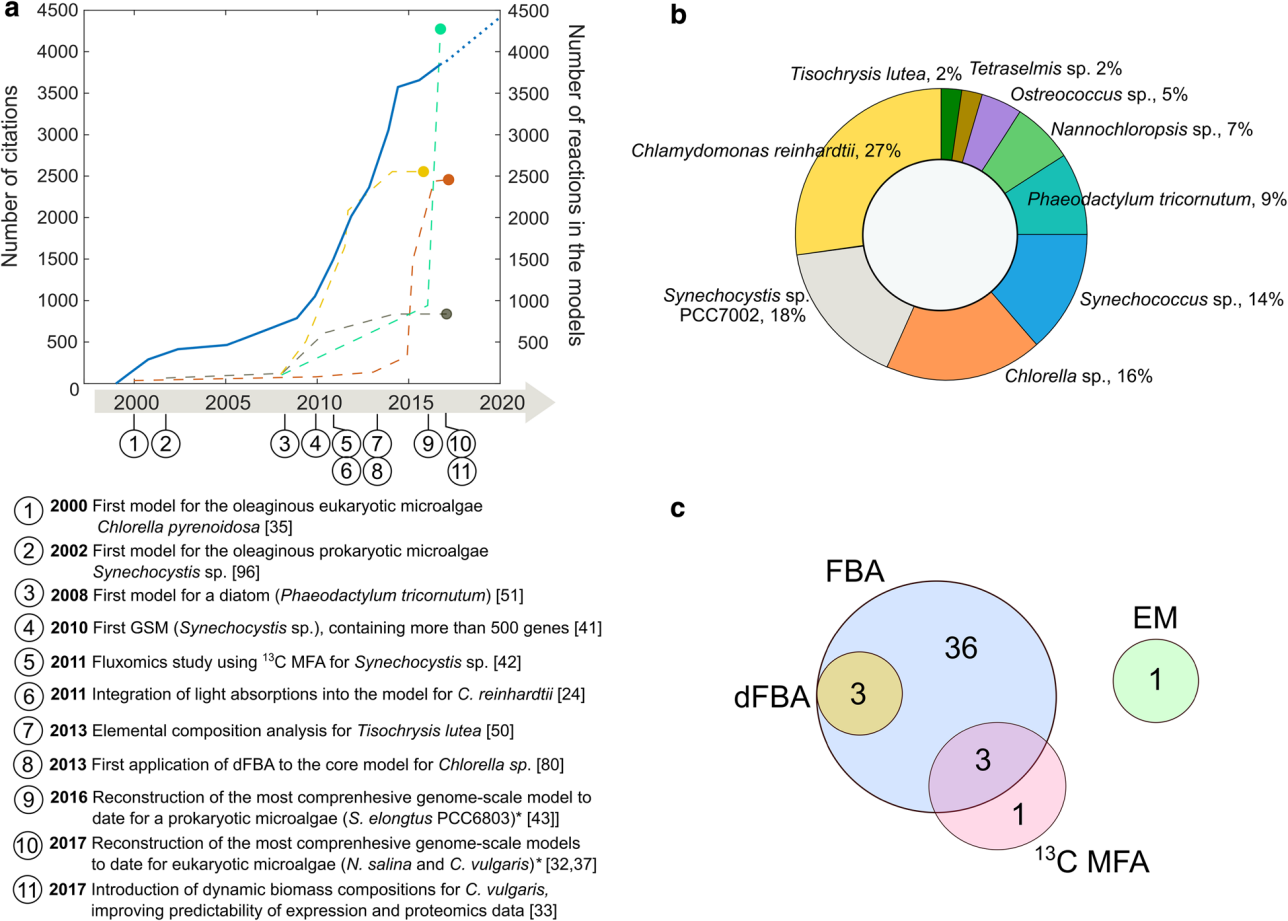
Key developments in constraint-based metabolic modeling of oleaginous microalgae. **a** Cumulative number of citations for all 44 publications related to “Metabolic Modeling of Oleaginous Microalgae and Cyanobacteria” (blue line) and conservatively estimated future citations (blue dotted line). Dashed lines represent the number of reactions per model for *Chlamydomonas* (yellow), *Synechocystis*, and *Synechococcus* (gray), *Chlorella* (orange), *Phaeodactylum* (green). **b** Breakdown of the total number of publications by microorganism (percentage) highlights the importance of model organisms such as *Synechocystis*, *Synechococcus*, *Chlorella*, *Chlamydomonas*, and *Chlorella*. **c** Frequency of metabolic modeling approaches used to solve models for oleaginous microalgae: flux balance analysis (FBA), followed by ^13^C metabolic flux analysis, dynamic flux balance analysis (dFBA), and elementary modes (EM)

Genome-scale metabolic models (GSMs) are a mathematical representation of all the available biochemical and genomic information about a specific organism. GSMs have extensively been used to guide strain engineering designs by optimizing biochemical processes within an organism [33]. The reconstruction of a metabolic network can start de novo by identifying and adding reactions one by one, or it can be initiated by the creation of a draft reconstruction based on sequence homology to another related organism [33]. As of May 2018, 44 metabolic models for oleaginous microorganisms have been reported. Details about characteristics of available models are summarized in Table 1. The highlights of milestones in metabolic modeling of oleaginous microalgae are shown in Fig. 1. While the first models for oleaginous microorganisms contained only core reaction, reaction size and complexity increased significantly over time (Fig. 1).

**Table 1:** Characteristics of current metabolic models of oleaginous microalgae

Organism	Metabolic model (ID)	Analysis	Genes	Reactions	Metabolites	Compartments	Citations [references]
*Chlamydomonas reinhardtii*	GSM	–	1069	–	–	–	143 [19]
*Chlamydomonas reinhardtii*	GSM	–	–	1500	1200	–	53 [20]
*Chlamydomonas reinhardtii*	GSM	FBA	–	484	458	3	292 [23]
*Chlamydomonas reinhardtii*	GSM	FBA	–	259	–	10	82 [24]
*Chlamydomonas reinhardtii*	GSM (AlgaGEM)	FBA	2249	1725	1862	4	96 [25]
*Chlamydomonas reinhardtii*	GSM (*i*RC1080)	FBA	1080	2190	1068	10	231 [26]
*Chlamydomonas reinhardtii*	CM	FBA	–	280	278	–	47 [27]
*Chlamydomonas reinhardtii*	GSM	FBA	–	160	164	2	100 [28]
*Chlamydomonas reinhardtii*	GSM	FBA	–	280	278	0	12 [29]^a^
*Chlamydomonas reinhardtii*	GSM (*i*BD1106)	FBA	1106	2445	1959	10	10 [30]^b^
*Chlamydomonas reinhardtii*	GSM (*i*Cre1355)	FBA	1355	2394	1133	10	12 [21]
*Chlamydomonas reinhardtii*	GSM	FBA/^13^C MFA	–	139	–	3	2 [22]
*Chlorella protothecoides*	CM	^13^C MFA	–	24	19	0	83 [34]
*Chlorella protothecoides*	GSM	FBA/^13^C MFA	461	272	–	4	0 [31]
*Chlorella pyrenoidosa*	CM	MFA	–	67	–	0	258 [35]
*Chlorella* sp.	CM	dFBA	–	114	161	–	31 [79]
*Chlorella variabilis*	GSM (*i*AJ526)	FBA	526	1455	1236	5	10 [91]
*Chlorella vulgaris UTEX 395*	GSM (*i*CZ843)	FBA	843	2294	1770	6	14 [32]
*Chlorella vulgaris UTEX 396*	GSM (*i*CZ946)	dFBA	946	2294	1770	6	2 [33]^c^
*Nannochloropsis gaditana*	GSM (*i*RJ1321)	FBA	1321	1918	1862	4	1 [38]
*Nannochloropsis salina*	GSM (*i*NS934)	dFBA	934	2345	–	10	4 [37]
*Nannochloropsis* sp.	GSM	FBA	383	987	1024	6	0 [36]
*Ostreococcus lucimarinus*	GSM	FBA	–	964	1100	2	38 [49]
*Ostreococcus tauri*	GSM	FBA	–	871	1014	2	38 [49]
*Phaeodactylum tricornutum*	GSM	–	151	88	–	5	289 [51]
*Phaeodactylum tricornutum*	GSM	FBA	–	–	–	2	12 [52]
*Phaeodactylum tricornutum*	GSM	FBA	607	849	587	6	27 [53]
*Phaeodactylum tricornutum*	GSM (*i*LB1027)	FBA	1027	4456	2172	6	24 [54]
*Synechococcus elongatus PCC7942*	GSM (*i*JB785)	FBA	785	850	768	7	13 [78]
*Synechococcus* sp. *PCC 7002*	GSM (*i*Syp611)	FBA	611	552	542	2	39 [92]
*Synechococcus* sp. *PCC 7002*	GSM (*i*Syp708)	FBA	708	646	581	2	39 [93]
*Synechococcus* sp. *PCC 7002*	GSM (*i*Syp821)	FBA	821	792	777	3	3 [94]
*Synechococcus* sp. *PCC 7002*	GSM (*i*Syp728)	FBA	728	742	696	7	22 [95]
*Synechocystis* sp. *PCC 6803*	CM	^13^C MFA	–	29	–	–	181 [96]
*Synechocystis* sp. *PCC 6803*	CM	FBA	–	70	46	2	165 [39]
*Synechocystis* sp. *PCC 6803*	CM	FBA	–	43	–	–	43 [40]
*Synechocystis* sp. *PCC 6803*	GSM	FBA	–	380	291	6	159 [41]
*Synechocystis* sp. *PCC 6803*	GSM	FBA	669	882	790	2	113 [44]
*Synechocystis* sp. *PCC 6803*	GSM (*i*Syn811)	FBA	811	956	911	2	59 [43]
*Synechocystis* sp. *PCC 6803*	GSM	FBA/^13^C MFA	–	493	465	2	51 [42]
*Synechocystis* sp. *PCC 6803*	GSM (*i*JN678)	FBA	678	863	795	3	206 [46]
*Synechocystis* sp. *PCC 6803*	GSM	FBA	677	759	601	6	143 [45]
*Tetraselmis* sp.	GSM	FBA	2249	1725	1862	4	2 [47]^d^
*Tisochrysis lutea*	CM	EM	–	157	162	2	2 [50]

Metabolic models are classified into two different groups: Genome*-*scale metabolic models (GSM) and core models (CM). The analyses were classified in: flux balance analysis (FBA), dynamic FBA (dFBA), elementary modes (EM), metabolic flux analysis (MFA), MFA using ^13^C tracer (^13^C MFA), and their combinations

^a^Modified the metabolic model of *C. reinhardtii* from Cogne et al. [27]

^b^Modified the metabolic model of *C. reinhardtii* from Chang et al. [26]

^c^Used the genome-scale model of *C. vulgaris* from Zuñiga et al. [32]

^d^Used the genome-scale model of *C. reinhardtii* from Dal’Molin et al. [25] with constraints for *Tetraselmis* sp.

**Fig. 2:**
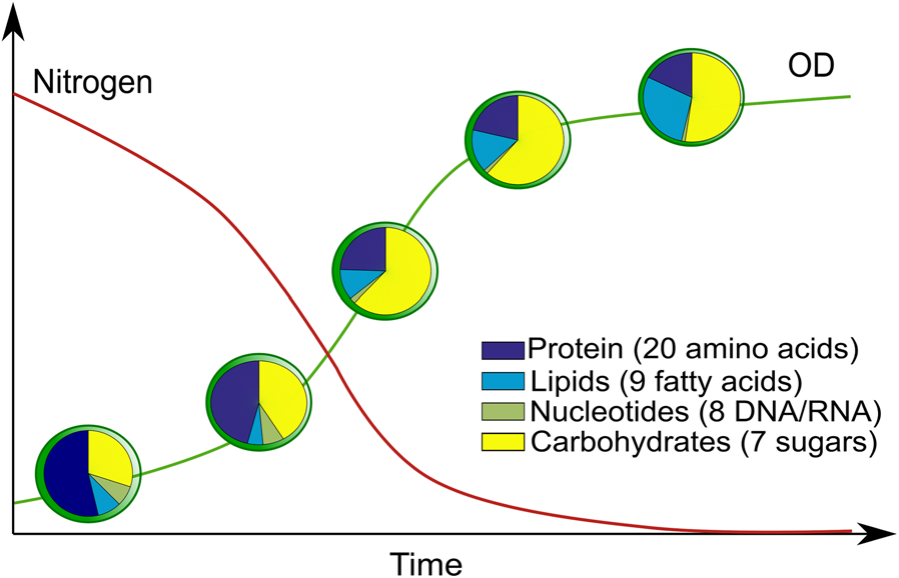
Changing biomass composition (*Chlorella vulgaris*) in response to nitrogen depletion determined over time. While available nitrogen (red line) decreases and optical density (OD, green line) increases over a growth course, the microalga accumulates storage compounds. Accumulation of storage compounds, such as lipids and carbohydrates, leads to a reduction of total protein. Data collected from [32]

The first GSMs for oleaginous microalgae were reconstructed for *Chlamydomonas reinhardtii* [19] and *Synechocystis* sp. [41]. Reconstructing a GSM model requires high-quality information on genome sequence, gene function, and metabolism [58–60]. Manual curation is required to improve the accuracy of the model. This curation process is very time and labor intensive, often spanning weeks to months before completion. To facilitate rapid model generation, automated pipelines, such as ModelSEED [61] and PATRIC [62], have been made publicly available. ModelSEED and PATRIC are reconstruction tools based on subsystems annotation, in which metabolic networks are decomposed into subsystems and analyzed individually. Both tools are based on RAST (Rapid Annotations using Subsystems Technology) that compares the genome sequence with existing information from phylogenetic neighbors [63]. However, it has to be noted that reconstructions created by automated tools are prone to errors and special attention must be directed toward quality control and quality assurance (QC/QA) tests, in particular with regard to mass balance and energy production without input [57, 64]. Automatically and semi-automatically reconstructed models thus require intensive manual curation before detailed and accurate predictions can be made. Figure 2a compiles the number of core and genome-scale models created for oleaginous photosynthetic microorganisms reported to date.

All GSM models can be expressed as a general mass balance, which includes every metabolite being produced or consumed within the network in its respective reaction. This mass balance takes the form shown in Eq. (1):

1 $$\begin{array}{*{20}c} {\frac{\text{d}}{{{\text{d}}t}}C = \left[ \varvec{S} \right]v.} \\ \end{array}$$

The vector *C* represents the instantaneous concentration of metabolites inside the cell, the vector contains all reaction rates and the matrix represents the stoichiometric information about reactions and participant metabolites. The stoichiometric matrix is a shared requirement among all constraint-based flux analysis approaches. Each column of this matrix contains the stoichiometric coefficients of a compound for all included reactions. In a similar fashion, each row represents the coefficients of all metabolites that take part in a single reaction [65]. An *m* number of metabolites would render the *S* matrix of *m* × *n* dimensions, with *n* always greater than *m*.

The rectangular nature of the S matrix is one of the most important obstacles to overcome when working with metabolic networks and is easily seen when taking into account that for *m* number of metabolites, there are *m* change rates inside vector *C*, *m* transport rates, and *p* intracellular rates that are unknown. The system of equations then comprises only *m* mass balances and as many as *n *= *2m *+ *p* variables [66]. This system indetermination is what has given birth to several different approaches to metabolic modeling, which are discussed below. For system determination to be achieved, the measurement of a total of *m *− *n* variables would be required. Large metabolic networks contain degrees of freedom that can amount to several hundreds. Therefore, the so-called core models, focusing on central metabolism, have been developed. These core models are used in metabolic flux analysis, such as the ^13^C-MFA, i.e., fluxomics. However, it is currently computationally infeasible to use large and compartmentalized metabolic networks for fluxomics analysis. Due to this, metabolic engineers have simplified the problem by transforming Eq. (1) into an optimization problem using an objective function and a defined set of constraints [65]. The definition of constraints results in a solution space, which delimits all possible functional states of a reconstructed network and a set of permitted phenotypes [67]. Metabolic models account for three types of constraints [65, 67]: (a) physico-chemical, which are based on conservation laws of mass and energy, dependency of reaction rates on biochemical loops and thermodynamics; (b) environmental, such as availability of nutrients, electron acceptors, and other external conditions (e.g. photon uptake); and (c) regulatory, including enzyme composition and performance, which helps to contextualize gene-related information, such as expression data and accurate gene–protein–reaction associations [68].

In phototrophic organisms, some physicochemical constraints are decided upon by following thermodynamic limits, regarding direction, reversibility or non-reversibility of reactions, which can be determined by calculating the Gibbs free energy. Environmental constraints are usually based on measured experimental values of light quality, and nutrient and substrate uptake rates. Some regulatory constraints are those used in a study by Levering et al., in which the GSM of the diatom *Phaeodactylum tricornutum* was employed to capture the response to varying environmental conditions due to a transcriptional regulatory network [69]. Despite this, there are still too many variables to account for in the dynamic system. Various approaches to analyze the metabolic network of oleaginous microalgae are discussed below.

### Flux balance analysis (FBA)

Most metabolic modeling studies involving oleaginous microalgae have been using FBA for simulation. A few other approaches have been used as an alternative or complement, such as ^13^C-MFA [22, 31, 34, 42] or EM [50]. Figure 1b, c highlights existing models and methods used to interrogate these models. Currently, large-scale metabolic networks are analyzed mainly in silico using FBA. Analysis of dynamic data obtained by experimentally intensive strategies like ^13^C-MFA rely on simplified metabolic models, e.g., representing only central metabolism [22, 31, 34, 42].

FBA refers to the application of linear programming to analyze fluxes under balanced metabolite conditions [65]. This statement is based on two assumptions: first, the cells are in steady state and, second, all cells have a general objective while growing. The first assumption simplifies the system significantly by neglecting all transient behavior of the metabolite concentrations, thus yielding Eq. (2). The elimination of all the unknown concentration change rates inside is mathematically convenient, but forces the system, i.e., a culture flask or bioreactor, to theoretically exist in a steady state.

2 $$\begin{array}{*{20}c} {\left[ \varvec{S} \right]v = 0 } \\ \end{array}$$

The second assumption of an objective function in the model implies that all cells grow with a specific objective, which is the same for every cell during the calculation time. The most widely used objective function for FBA is the maximization of biomass production, which implies that the organism has evolved sufficiently to have the optimal arrangement of fluxes so that its growth will be maximized. While this assumption is likely correct for certain microorganisms, it is not universally applicable [70]. For example, under nutrient-deficient conditions the objective of a cell might not be biomass production, but rather the optimization of the production rate of storage compounds for later use. In a similar way, we know that phenotypic states vary in accordance with the growth phase or environmental conditions (Fig. 2), especially those that exhibit a dynamic biomass composition, such as phototrophs [71–73] and yeast [74]. Thus, time-specific biomass compositions are needed for light–dark cycles, considering degradation of storage pools during dark periods. This is of particular interest for the production of biofuel precursors. Furthermore, maximization of carbon uptake rate as CO_2_ has been proposed as a suitable objective function for autotrophic modeling during the light period [32]. FBA has proven to be useful and to reproduce overall experimental behavior in silico, although a true steady state is hardly encountered in experimental settings [58]. Its versatility and the accurate reproducibility of experimental results under several culture conditions make FBA one of the most widely used methods for metabolic modeling [75].

### Biomass objective function

The biomass objective function (BOF) is a broadly used modeling reaction, which drives the supplemented resources across the metabolic network to produce all known cellular components in the model (such as amino acids, nucleotides, fatty acids, carbohydrates, vitamins, ions, and cofactors). Maximizing the BOF allows simulating growth rate and the yield of carbon source to biomass (henceforth referred to as biomass yield). The BOF can be determined from the genome sequence [59] or through experimentation. Both approaches have been successfully applied, especially for prokaryotic microorganisms. However, when microorganisms have been subjected to non-optimal conditions, such as extreme temperatures, pH, or limited nutrient concentrations, a single BOF is often not suitable to predict experimental data successfully [70, 76]. For these cases, auxiliary objective functions have been proven necessary, such as minimization of ATP production, substrate uptake rate, or redox potential production rate [70].

There are several levels of refinement of the BOF [77], but it generally consists in the definition of a set of metabolites which compose the biomass. The set can be composed of just one reaction yielding a hypothetical compound called “biomass” or could otherwise be refined up to building blocks or biomass components (carbohydrates, lipids, proteins, DNA, RNA, pigments, etc.) [78]. The BOF of manually curated metabolic models of oleaginous microorganisms often accounts for hundreds of metabolites as part of the lipid metabolism, because of lipids being the primary target for biofuel production in these organisms. Lipid chain fatty acids (14:0, 16:1, 18:1, 16:2) are usually summarized as triacylglycerols (TAG), monogalactosyldiacylglycerols (MGDG), etc., representing the entirety off all lipids in the organism. Accurate BOF composition has enabled the improved prediction of phenotypic states. It has been claimed that constrained BOF furthers the predictability of experimental nutrient- and light-limited conditions [33]. In some cases, the BOF has been complemented by a two-step optimization approach with minimization of uptake rates. In autotrophic growth conditions, minimization of light uptake (photons) has been employed but no significant improvement of the growth rate prediction has been obtained [23, 39]. In the same way, minimization of carbon source substrate uptake rate has been utilized for heterotrophic growth [25, 47]. As alternatives, minimization of flux magnitudes across the network was used for *P. tricornutum* [51, 54], maximization of ATP yield [28], and minimization of ATP demand [24] for *C. reinhardtii*, and maximization of hydrogen production rate for both *C. reinhardtii* [25] and *Synechocystis* sp. [40].

### Dynamic FBA

Overcoming the steady-state assumption of standard FBA is vital for the modeling of highly dynamic systems, which are characteristic of photosynthetic microorganisms [33, 37, 79]. These organisms have evolved under cyclic light/dark conditions, which require switching between different phenotypic states. During light periods, inorganic carbon is fixed into storage carbon compounds, such as carbohydrates and lipids, which are consumed in the dark period to accommodate vital cell functions. The storing-for-later behavior results in a dynamic biomass composition that can change during the light period (hours) or along the course of growth (days). In the case of *C. vulgaris* and other phototrophs, it has been shown that the biomass composition is also dependent on nitrogen availability (Fig. 2). Since FBA is used under a steady-state assumption, it is virtually disqualified for its use in the aforementioned cases. On the other hand, not including this assumption would add a set of ordinary differential equations to the problem, yielding a differential–algebraic system. To solve this, a dynamic FBA approach was proposed using either a dynamic optimization approach (DOA) or a static optimization approach (SOA) [80].

The DOA calculates the time profiles of fluxes and metabolite concentrations by solving the optimization problem over the entire time span of interest, running the calculation only once. The dynamic system is transformed into a non-linear programming problem (NLP) by parameterizing the differential equations through the method of orthogonal collocation on finite elements, described by Cuthrell and Biegler [81]. The BOF is then rewritten as a weighted average of the instantaneous and the terminal objective functions and is subjected to the system of differential equations along with the constraints. The SOA approach, on the other hand, solves the optimization problem multiple times, once for each time interval. At the end, an integration of the set of instantaneous rates of change over the interval is carried out for the calculation of metabolite concentrations.

Experiment-based BOF constraints are an alternative method to simulate dynamic metabolic behavior [33]. Changes in the BOF influence the state of the metabolic network, thus directly affecting predictions. This approach improved the accuracy of flux prediction by considering measurements over the course of growth under autotrophic and heterotrophic conditions in *Chlorella vulgaris*. The time series flux distributions accurately simulate 75% of expression and proteomics data collected over the course of growth, including allosteric reactions and multi-subunit enzymes. This approach also enabled the determination of the net content of nitrogen pools at each condition [33]. When an experimental determination of metabolites constituting the BOF is not feasible, unsteady-state methods, such as unsteady-state FBA (uFBA), can be applied. These unsteady-state methods operate with a limited number of measured metabolites. uFBA was recently developed and applied to study heterotrophic microorganisms [86], but uFBA would be a promising approach for the analysis of photosynthetic microorganisms.

### Unsteady-state FBA

The aim of uFBA is to calculate internal flux distributions from existing time-course data, e.g., target metabolomics data. These datasets typically contain information about several (five to ten) metabolites such as glycerol, ethanol, and acetate. It is necessary to determine the rate of change of these metabolites from the experimental data and to include these rates in the system of equations [82]. Ideally, all rates of change would be known and the uFBA could be run as a series of standard FBA methods. Since this is often not feasible, all immeasurable variables are assumed to be, initially, under steady-state conditions as well as under a closed system assumption, i.e., with no possibility of transport inside or outside the cell. Elimination of this amount of transport reactions can often over-determine the system and requires further conditioning. A “metabolite node relaxation” algorithm has been deployed that assigns sink reactions to unmeasured variables to allow for their accumulation or depletion. The algorithm is based on optimizations that find the minimum number of sink reactions that are necessary while keeping the model computable [86].

### Metabolic flux analysis (MFA)

MFA is an alternative to FBA which also assumes a steady-state mass balance [83]. When working with small enough metabolic networks, it is possible to measure or define enough numbers of internal or external fluxes to determine the algebraic equation system. For this strategy, Eq. (2) is rewritten by decomposing the matrix and the vector into the measurable (known) and the immeasurable (unknown) fluxes, as shown in Eq. (3).

3 $$\left[ \varvec{S} \right]_{u} v_{u} + \left[ \varvec{S} \right]_{m} v_{m} = 0$$

The larger the metabolic network, the more the fluxes are necessary to measure for system determination. Therefore, metabolic networks of several hundred reactions require measurements of internal fluxes for most of the fluxes, e.g. by ^13^C labeling [22, 31, 42].

### Elementary modes (EM)

EM is based on the calculation of all the solutions of the system in Eq. (2) in the allowable flux space, restricting the solution with a thermodynamic constraint and a non-decomposability constraint [84]. The latter renders each solution an elementary flux mode, which means it is a unique and minimal set of reactions. These sets can be rewritten into macroscopic reactions, thus reducing the degrees of freedom exhibited formerly by Eq. (2). Often, EM is combined with core genome-scale metabolic models to provide energetic efficiencies and optimal flux distributions [84, 85]. The use of EM analysis (Fig. 1c) has declined over the last years, in part due to the rapid development of omic tools applied to sequencing, which enables generating genome-scale metabolic network reconstructions based on complete genome sequences.

## Lessons learned from metabolic modeling of oleaginous phototrophs

Advances in modeling of microalgae are in part due to the availability of extensive omic datasets. Having full genome sequences available was crucial for generating the initial genome-scale metabolic models for the microalgae *Chlamydomonas* [23, 26] and opened the possibilities of creating additional algae models based on homology [32]. Fluxomic data has played a major role in elucidating central carbon metabolism of microalgae (e.g., *C. protothecoides* [34], *C. reinhardtii* [22], and *Synechocystis* sp. [42]). This tool has also served as a validation tool for GSM models [30]. In addition, metabolomics or transcriptomics have been used in context of the model to study and optimize biochemical pathways of industrial interest [86]. In this section, we describe different approaches to reconstruct and simulate metabolic models for oleaginous microalgae to increase growth and lipid content and improve bioproduction.

### Growth conditions

Several microalgae are able to grow as autotrophs, heterotrophs, or mixotrophs. Some metabolic pathways are only active under certain growth modes, including the oxidative/reductive pentose phosphate pathway [22, 23, 27, 39, 40], Calvin cycle, and presumably the glyoxylate shunt [39]. Hence, differential mathematical models are necessary for correct prediction for each growth condition, requiring unique stoichiometric matrices and biomass formation equations. The study and prediction of phenotypes dependent on growth conditions is perhaps the most studied aspect regarding in oleaginous microalgae. Currently, the models accurately predict growth with hundreds of different nitrogen and carbon sources [30, 32]. Furthermore, most models are able to reproduce more than one growth mode, including mixotrophy in the models *i*CZ843 [32], *i*CZ946 [33], *i*RJ1321 [38], *i*RC1080 [26], AlgaGEM [25], *i*NS934 [37], *i*LB1027 [53], and a model for *Nannochloropsis* sp. [36].

Experimentally, highest biomass yields have been reported for autotrophic conditions, while lowest were obtained under heterotrophic growth in *P. tricornutum* [53], *Synechocystis* sp. [39], *C. reinhardtii* [23], and *Chlorella* sp. [79]. Mixotrophic growth, as a kind of hybrid condition, has shown biomass yields falling between ones observed for autotrophic and heterotrophic. However, an exception is the study of Navarro et al. [40], in which a mixotrophic biomass yield (92%) higher than the autotrophic one (60%) was predicted as reported for *Synechocystis* sp. The constraints regarding growth conditions directly affect the way carbon is distributed across the metabolic network, and thus the biomass yield and production rate. So, it is crucial to determine and adjust these constraints if needed for each growth condition. For example, under autotrophic growth the biomass yields have been reported to be close to 100%, since no carbon is lost in the process [23, 39, 44, 53, 79], thus experimental measurements can be used directly. On the other hand, under heterotrophic growth conditions a significant carbon loss as CO_2_ in oleaginous microalgae has been reported to vary between 37% [39] to 40% for *Synechocystis* sp. [40], 50% for *C. reinhardtii* [23] and 50.5% for *Chlorella* sp. [79] as a result of the carbon input flux being lost as CO_2_ due to energy production through the TCA cycle and the oxidative pentose phosphate pathway (PPP) [23, 39, 44, 53, 79]. Mixotrophic biomass yields tend to be higher than under heterotrophy, since part of the released CO_2_ is fixed once again [39]. Reported net biomass yields are therefore around 92% (*Synechocystis* sp.), 100% (*C. reinhardtii*) and 80% (*Chlorella* sp.) assuming a closed system.

### Light conditions

Since light directly impacts microalgae growth and behavior, efforts have been made to define the quality and quantity of light constraints in metabolic models [29, 37]. Models can be significantly improved by considering a more realistic light uptake mechanism, since correctly defined constraints regarding light-driven reactions allow for the assessment of light influence on carbon allocation. Chang et al. [26] proposed dividing the total light spectrum into effective spectral bandwidths, each of which had an associated effective bandwidth coefficient. These coefficients, along with the activity spectra of light-driven reactions, allowed for the correct calculation of flux distribution along these reactions, taking into account that phototrophic organisms are strongly affected by the nature of the incoming light. Manually curated models account for reactions to simulate light sources, such as solar, incandescent, fluorescent, metal halide, high-pressure sodium, red and white LED. High-resolution light phenomena in the model enables to determine the most suitable irradiance conditions for increased growth and lipid productivities. However, it has been shown that the bandwidth coefficient varies from microorganism to microorganism, as well as with culture size and growth vessels used, for example flasks, pilot, or industrial-scale vessels. Therefore, microorganism-specific refining for light uptake modeling in the GSM models will be needed for further improvement [39].

### Intracellular pools

Although metabolic modeling has focused on simulating the intracellular environment of a cell under steady state, allowing the accumulation of certain metabolites in pools has proven necessary for the correct prediction of phenotypic states [60, 87]. Metabolite pools can play an important role in the regulation of reactions, since different pathways find themselves interconnected by common metabolite collections. Target metabolomics data have been used successfully to constrain the metabolic model of *Chlorella* and determine the pool size of nitrogen [33]. The determination of the pool size was achieved by scanning the nitrogen uptake rate while fixing experimentally determined biomass compositions over the course of growth. Thereafter, nitrogen pool concentrations were computed by integrating the predicted nitrogen uptake rates necessary to meet the required biomass composition each time. Other target examples are energy-dependent and energy-replenishing processes which are coordinated by the ATP, ADP and AMP pools [87] as well as nitrogen and chrysolaminarin pools in *P. tricornutum* [54].

### Compartmentalization

Eukaryotic microalgae contain different organelles (e.g., cytosol, mitochondria, nucleus, endoplasmic reticulum, glyoxysome, chloroplast, Golgi apparatus, vacuole, thylakoid, eye spot, and the cell wall). The exact compartmentalization is species dependent. Accurate annotation of proteins and compartmentalization in the model is necessary for maximizing information content and gaining detailed knowledge about microalgae metabolism. Flux distributions highly depend on the model’s capability for metabolic exchange prediction between organelles. Careful manual curation of these models and delimitation of capabilities while adding reactions and reconstructing eukaryotic models in an automatic matter is thus crucial to achieve maximal predictability [63].

The example of nicotinamide adenine dinucleotide phosphate (NADPH) production in eukaryotic microalgae highlights the importance of compartmentalization. The PPP plays the role of producing NADPH in the cytosol, while the electron transport chain (ETC) is in charge of producing it in the chloroplast. Non-compartmentalized models can predict that the entire NADPH demand is supplied by the ETC, rather than PPP supplying NADPH demand outside the chloroplast. This issue was encountered in the first metabolic model of an oleaginous microalgae *C. pyrenoidosa* [35]. While the model can simulate central carbon metabolism in general, it cannot predict detailed engineering targets since information about where fluxes take place is not available.

Early metabolic models were focused on the reconstruction of core algae models, which were later expanded to include genome-scale information (Table 1) [19, 20, 35, 49, 50]. The least compartmentalized model included only the chloroplast and cytosol, to uncouple the NADPH consumption/production of the Calvin cycle and the PPP [28]. More refined models now account for the mitochondria, thylakoid lumen, glyoxysome (peroxisome), extracellular environment, nucleus, Golgi apparatus, endoplasmic reticulum, vacuoles, and the cell wall [21, 24, 26, 30, 36, 37].

### Modeling lipid production

Phototrophs produce several different kinds of lipids, including tri- and diglycerides, phospho- and glycolipids and hydrocarbons [14]. More specifically, lipid production in oleaginous microalgae includes triacylglycerol (TAG), phosphatidylglycerol, phosphatidylinositol, phosphatidylethanolamine, sulfoquinovosyldiglycerol, MGDG, digalactosyldiglycerol, and phosphatidylcholine. TAG alone can accumulate from 20 to 60% of dry cell weight in some species like *C. vulgaris* [32]. Modeling and gaining insight into the increased lipid content of microalgae has been the object of several studies. Most of these studies have investigated the effect of nitrogen depletion [21, 33, 34, 37, 38, 42, 48, 54], while others have studied the influence of low CO_2_ and low light [54] on increasing overall lipid content. A study of *Nannochloropsis gaditana* reported increased lipid productivity under mixotrophic growth conditions [38].

When microalgae are subjected to nitrogen depletion conditions, carbon flux is drawn away from photosynthetic pathways as cells shift into the stationary phase and begin to store carbon as starch and lipids. This phenomenon and its effect on biomass composition are displayed in Fig. 2, in which a general trend of biomass composition evolution as a function of time and nitrogen availability is presented. Under these non-optimal culture conditions, microalgae shift the central carbon flux from biomass production to the production of storage compounds. As a result, the growth rate is decreased because carbohydrates and/or lipid are accumulated under stress conditions [40]. *C. protothecoides* was reported to redirect 81% of the input carbon flux toward fatty acid synthesis, but as a consequence decreased its growth rate by 30% [34]. In a similar way, *C. reinhardtii* was found to accumulate TAG when faced with nutrient limitation, but its growth halted after 8 h of cultivation [22]. Interestingly, Lim et al. [47] reported downregulation of TAG biosynthesis for *Tetraselmis* sp. after 24 h, though TAG accumulation continued after this time point. The authors claimed this to be a result of decreased lipid degradation rather than lipid production.

## New insights into the central carbon metabolism of microalgae

**Fig. 3:**
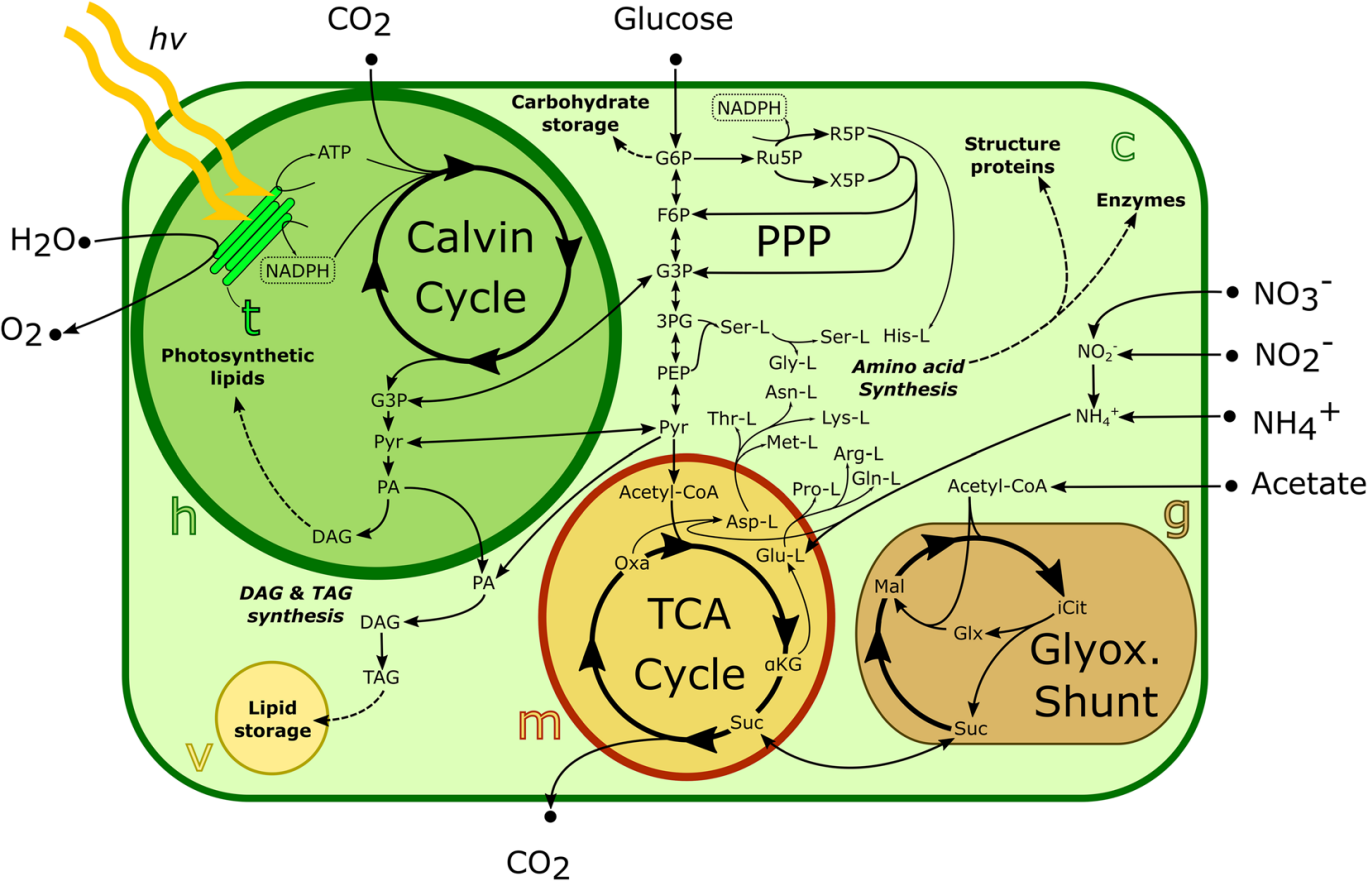
Central metabolism in eukaryotic microalgae. The main compartments of active metabolism are shown, i.e., the chloroplast (h), thylakoid lumen (t), vacuole (v), mitochondrium (m), glyoxysome (g), and cytosol (c)

Most studies on oleaginous microalgae have focused on the central carbon metabolism and revealed new findings about glycolysis, PPP, TCA cycle, and the Calvin cycle. Figure 3 shows the most important metabolic pathways in microalgae and how they are linked to lipid metabolism. FBA has been used to study genome-wide fluxes through the metabolic network under mixotrophy, heterotrophy, and autotrophy. While most studies coincide in their assessment of functionality and fluxes in central carbon pathways, other pathways such as the glyoxylate and ornithine shunt are still not well understood, and modeling results are often not consistent between different studies and organisms [22, 23, 25, 31, 34, 39, 40, 42, 79]. As a general rule, significant carbon flow through the TCA cycle has been reported under heterotrophic conditions, which demand catabolism of external organic compounds, contributing to the reduction of flux through the electron transport chain (ETC) and the Calvin cycle [23, 32, 35, 38]. During heterotrophic growth, most microalgae prefer glucose as carbon and energy source (Fig. 3). Other microalgae, such as *C. reinhardtii*, are only capable of assimilating two-carbon organic compounds, like acetate [22]. When glucose enters the cytosol, its fate can either be oxidation via glycolysis to pyruvate, oxidation via PPP to ribose 5-phosphate or transformation into storage compounds (lipids, glycogen and starch) [88]. In microalgae, acetate coming from the extracellular environment can be converted in the glyoxysome to succinate through the glyoxylate shunt, which can be considered as a variation of the TCA cycle. Succinate, an important biosynthetic precursor that can be converted into oxaloacetate, from which phosphoenolpyruvate (PEP) can be synthesized by the enzyme PEP carboxykinase, and enter gluconeogenesis for carbohydrate or lipid synthesis [17]. Under autotrophic growth, the carbon source is inorganic (CO_2_) and the energy source is light. In the thylakoid lumen of eukaryotic microalgae, the ETC takes advantage of protons from light to store its energy in the form of ATP and NADPH, which are subsequently used to reduce CO_2_ molecules into triose phosphates (G3P) in the Calvin cycle. G3P can then be converted into glucose 6-phosphate (G6P), sucrose, starch and other sugars in the chloroplast.

### Tricarboxylic acid cycle

**Fig. 4:**
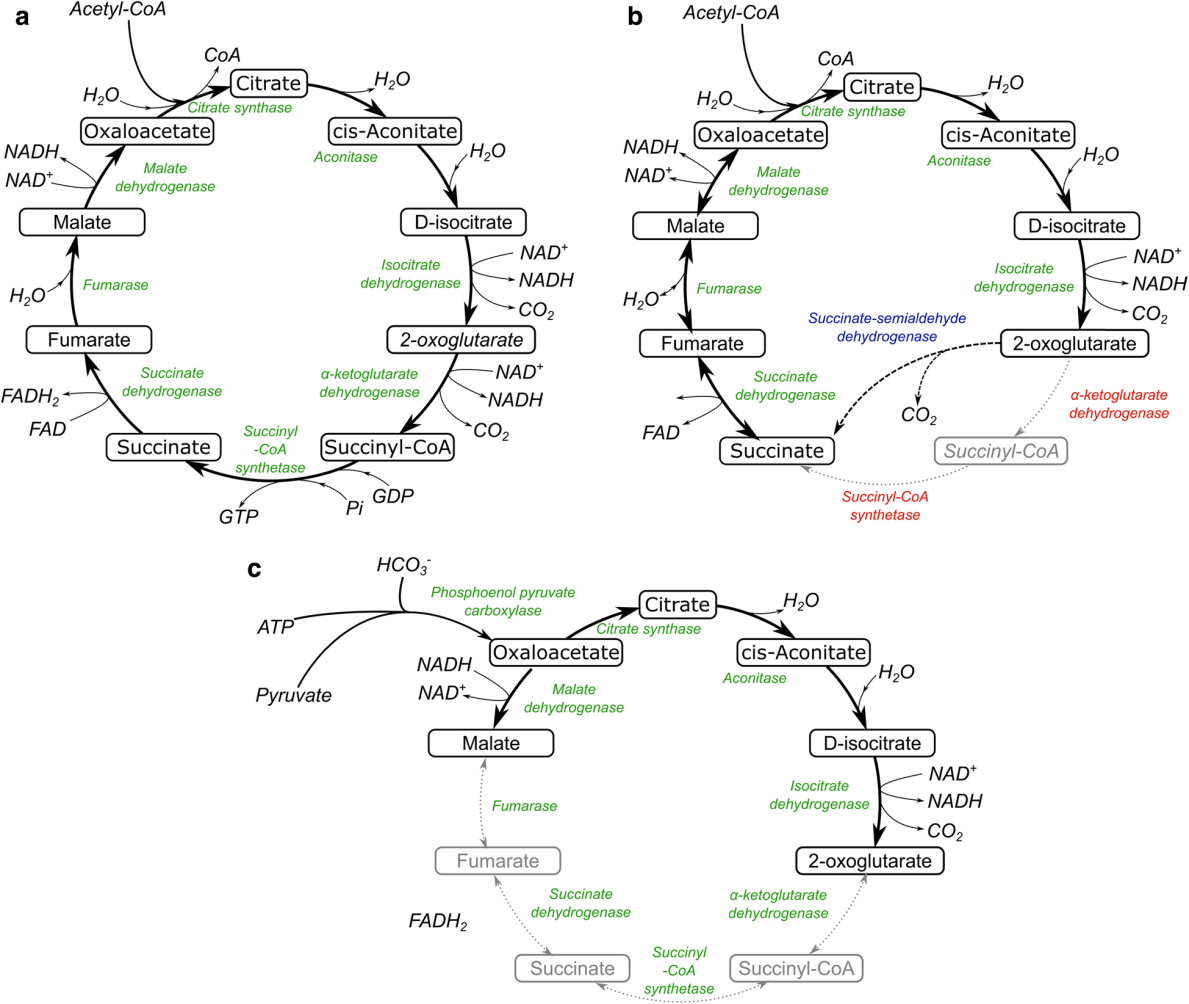
Variations of the TCA cycle in photosynthetic microorganisms. **a** Complete and fully functional TCA cycle. **b** TCA cycle observed in microalgae, such as *Synechococcus* sp., which lacks the enzymes α-ketoglutarate dehydrogenase and succinyl-CoA synthetase (enzymes highlighted in red). A bypass via succinate-semialdehyde dehydrogenase, as observed in *Synechocystis* sp., is shown in blue. **c** Split TCA cycle as reported for *C. reinhardtii* [30]. The two branches producing 2-oxoglutarate and malate for downstream biosynthesis. Oxaloacetate is provided via anaplerotic activity of phosphoenolpyruvate carboxylase in this split TCA cycle [46]

The TCA accounts for the highest carbon fluxes and number of active reactions under heterotrophic growth conditions [32, 35]. Under this mode, the percentage of the total carbon input flux into the TCA cycle was reported to be 35.6% in *C. reinhardtii* grown with acetate [22] and 18.7% in *C. protothecoides* grown with glucose. However, under autotrophic and mixotrophic conditions, only half of the activity has been reported [79], with only 8–10 out of 22 reactions carrying flux for both microorganisms [32]. The role of the TCA under these conditions shifts to the production of biosynthetic precursors rather than energy production. Figure 4 shows complete and possible bypass variations of the TCA cycle observed in different photosynthetic microorganisms. Cogne et al. [27] reported that under autotrophic growth the TCA cycle in *C. reinhardtii* was operating as two branches with production of 2-oxoglutarate on one end, and malate on the other, with an input through oxaloacetate via the anaplerotic activity of the phosphoenolpyruvate carboxylase (Fig. 4). Zero flux was found through the enzymes 2-oxoglutarate dehydrogenase, succinyl-CoA synthetase, and succinate dehydrogenase, since energy demands can be supplied by the PPP and the glyoxylate shunt. Other studies have also reported such similarities between prokaryotic and eukaryotic organisms [89, 90], in which prokaryotic microalgae, such as *Synechocystis* sp. and *Synechococcus elongatus*, do not possess a complete TCA cycle. These bacteria lack the α-ketoglutarate (2-oxoglutarate) dehydrogenase and succinyl CoA synthetase [17, 78]. Knoop et al. [41] have claimed a bypass via the succinate-semialdehyde dehydrogenase to compensate for the lack of 2-oxoglutarate dehydrogenase as shown in Fig. 4. The bypass replenishes intermediaries in the TCA cycle linked to lipids biosynthesis such as acetyl-CoA.

### Reductive/oxidative pentose phosphate pathway

The oxidative and reductive phases of the PPP serve different purposes. While the oxidative phase serves as a catabolic pathway for NADPH production from the oxidation of G6P, the reductive phase represents an anabolic pathway of biosynthesis of 5-carbon carbohydrates for synthesis of nucleic acid, coenzyme A, ATP, and other biomass and lipids biosynthetic precursors [79]. High flux through the oxidative PPP means that energy production is being used for maintenance rather than growth [35]. It has been reported that depending on the growth conditions, either one phase can appear active. However, for the diatom *P. tricornutum* a low flux through the whole PPP pathway was determined. The reduced flux is explained with this organisms’ ability to obtain 5-carbon carbohydrates via phosphopentose epimerase [53].

As a general result for non-compartmentalized models, energy-yielding oxidative PPP appears inactive during autotrophic growth, since the model predicts energy comes from the ETC in the form of NADPH rather than the dissimilatory pathways [35, 39]. As stated above, NADPH demand outside the chloroplast should be supplied by the PPP rather than the ETC. However, the compartmentalized models of Cogne et al. [27] and Boyle and Morgan [23] predicted inactivation of the oxidative PPP for *C. reinhardtii* under autotrophic conditions. In the latter study, cells were found to prefer indirect energy transport by taking G3P from the chloroplast to the mitochondria and degrading it to 3-phosphoglycerate (3PG), releasing both ATP and NADH [23]. Furthermore, the fact that *C. reinhardtii* uses acetate as a carbon source instead of glucose greatly affects its phenotypic behavior and flux distribution under heterotrophy. Since the input to the PPP is G6P, incoming acetate would have to be transformed through several reactions in the glyoxylate shunt to oxaloacetate and then to G6P (Fig. 3). For this reason, NADPH production in *C. reinhardtii* preferably takes place via the ETC under autotrophic growth, while it is produced mainly through the glyoxylate shunt under heterotrophic growth [22, 23, 31, 34, 35, 39–41, 79]. Limitation in the transport or consumption of G6P or 3PG can result in metabolite accumulation, leading to the synthesis of certain types of lipids. For example, *C. reinhardtii* produces mainly triglyceride lipids.

Apart from growth conditions, other external factors have been reported to alter the flux distribution through the PPP. Wu et al. [31] found that increased oxygen availability in *C. protothecoides* decreases the flux through the PPP and instead enhances flux through the TCA cycle, thus producing more energy and yielding more CO_2_. Moreover, increased synthesis of storage compounds under nitrogen-depletion conditions was shown to increase PPP fluxes due to increased demand of NADPH for biosynthesis [34].

### Glyoxylate shunt

The ability of the glyoxylate shunt of transforming acetyl-CoA into succinate for biosynthetic purposes renders it vital for the metabolism of acetate independent of its source, i.e. extracellular environment. However, the glyoxylate shunt has been found to be inactive under heterotrophic [31, 34, 79], autotrophic [39, 40, 79], or mixotrophic growth conditions [42] for various organisms, e.g., *Synechococcus* sp. In *C. reinhardtii* and *P. tricornutum*; however, the glyoxylate shunt has been reported to be active for all tested heterotrophic conditions [22, 23, 25]. The inactive glyoxylate shunt under autotrophic growth can be explained by the cell not taking up acetate from the environment, but rather synthesizing storage compounds, such as lipids and carbohydrates, that represent desirable bioproducts [40, 80].

### Calvin cycle

Reducing equivalents and ATP formed in the ETC under autotrophic conditions are used later in the Calvin cycle to produce triose phosphates (G3P) for further synthesis of carbohydrates, which can be assimilated or turned into backbone structures of lipids. During autotrophic growth conditions, the entire anabolic activity relies on the Calvin cycle. G3P is transformed into higher carbohydrate molecules, such as pentoses and hexoses, through the PPP and gluconeogenesis, respectively. Moreover, lipid and amino acid anabolism is dependent on pyruvate produced from the G3P [88]. It has been reported in green algae that the Calvin cycle fixes CO_2_ in the form of 3PG, which gets converted to dihydroxyacetone phosphate (DHAP) subsequently [79]. Naturally, the Calvin cycle is inactive in the dark. When microalgae are subjected to mixotrophic conditions, carbohydrate demand poses a competition between uptake of external organic carbon sources and the Calvin cycle (i.e., inorganic carbon uptake). In *C. reinhardtii* the majority of carbon flux was found to be directed toward the Calvin cycle, rather than glycolysis and TCA under mixotrophic growth [23]. The cyanobacterium *Synechocystis* sp. however, was found to be dominated completely by the organic carbon uptake before a specific threshold of light intensity was surpassed. After this verge of irradiance, rubisco-dependent carboxylation and oxygenation were increased immediately and all Calvin cycle reactions were activated [41].

## Conclusions

Great advances have been made in constraint-based modeling of photosynthetic microorganisms over the last two decades. Metabolic modeling has been proven critical for our understanding of complex metabolism in microalgae. Model-driven approaches have helped to identify boundaries for light and nutrient conditions as well as suitable genetic targets to increase lipid productivity. Metabolic models have progressed from core models to genome-scale metabolic models, which now include detailed compartmentalization and light uptake. Furthermore, the dynamic behavior and rapidly changing phenotypes due to changing environmental parameters are important traits of these organisms and have now been included in model simulations. Those recent extensions and improvements allow elucidating phenotypic behavior under different growth and culture conditions over time. In addition, these new models provide a high-quality standard for the improvements of existing metabolic models as well as for future reconstructions. Despite extensive efforts on refinement and manual curation of metabolic models, there are still open questions regarding the central metabolism and dynamic biomass composition in microalgae. Coupling metabolic modeling with fluxomic experiments can improve our knowledge of the activity of the glyoxylate shunt and ornithine shunt. Furthermore, time course-dependent expression datasets are needed to constrain and validate the models and to gain insight into the dynamics of metabolism. These datasets will enable to broaden the scope of the models and to elucidate missing transport reactions. Multi-omics dataset can also increase the predictability of carbon exchange and storage within the cell and guide improved production of desirable compounds in microalgae.

## Abbreviations

ADP: adenosine diphosphate
AMP: adenosine monophosphate
ATP: adenosine triphosphate
BOF: biomass objective function
C: metabolite concentration vector
CBFA: constraint-based flux analysis
CO_2_: carbon dioxide
CoA: coenzyme A
dFBA: dynamic flux balance analysis
DHAP: dihydroxyacetone phosphate
DNA: deoxyribonucleic acid
EM: elementary modes
ETC: electron transport chain
FBA: flux balance analysis
G3P: glyceraldehyde-3-phosphate
G6P: glucose-6-phosphate
GSM: genome-scale metabolic (model)
MGDG: monogalactosyldiacylglycerols
MFA: metabolic flux analysis
NADH: nicotinamide adenine dinucleotide
NADPH: nicotinamide adenine dinucleotide phosphate
NLP: non-linear programming
PEP: phosphoenolpyruvate
PPP: pentose phosphate pathway
QA: quality assurance
QC: quality control
RNA: ribonucleic acid
S: stoichiometric matrix
TAG: triacylglycerols
TCA: tricarboxylic acid (cycle)
uFBA: unsteady flux balance analysis
